# Duplex scan in patients with clinical suspicion of deep venous thrombosis

**DOI:** 10.1186/1476-7120-6-53

**Published:** 2008-10-20

**Authors:** Aguinaldo de Oliveira, Graciliano J França, Enrique A Vidal, Paulo SDB Stalke, Liz AV Baroncini

**Affiliations:** 1 Federal do Paraná University, Faculdade de Mecidina, Department of Surgery, Rua XV de Novembro 1299, Centro. CEP:80060-000 Curitiba, Paraná, Brazil

## Abstract

**Background:**

The incidence of deep venous thrombosis is 0.6/1000 habitants and when symptomatic its diagnosis by duplex scan has 100% sensitivity and 98% specificity.

**Objectives:**

The aim of this study was to evaluate the findings of the duplex scan in patients with clinical suspicion of deep venous thrombosis.

**Methods:**

239 consecutive outpatients (59.2 ± 18.6 years, 164 female) were evaluated by duplex scan.

**Results:**

According to symptoms 101 (42.3%) were related to the right lower limb; 113 (47.3%) to the left lower limb; and 25 were related to both lower limbs. Forty-eight patients presented a normal duplex scan. Venous thrombosis was found in 117 patients (0.49; CI 0.43–0.55): 75 with deep venous thrombosis (DVT), 22 with superficial venous thrombosis (SVT) and 20 with both DVT and SVT. Other pathologies were found in 74 patients. Among patients with DVT the most involved veins were below the knee. Among patients with SVT, 20 (47.6%) showed progression to the deep venous system: 9 (45%) by perforans veins; in 6 by saphenous-femoral junction (30%); and in 5 (25%) by saphenous-popliteal junction.

**Conclusion:**

Deep venous and superficial venous thromboses were diagnosed in 49% of cases. In 30.9% of cases, the duplex scan indicated other pathologies.

## Background

Deep venous thrombosis (DVT) is a frequent and potential fatal disease with estimated incidence of 0.6 cases per 1000 inhabitants/year in our environment; 0.8 cases per 1000 inhabitants/year in the USA; and 0.9 cases per 1000 inhabitants/year in Sweden [1]. The incidence of DVT is similar in males and females and increases dramatically with age from about 2–3 per 10000 person years at age 30–49 to 20 per 10000 person years at age 70–79 and around 40% of cases of DVT are idiopathic [2]. The diagnosis of symptomatic deep venous thrombosis (DVT) is well established using duplex scanning, with a sensitivity of 100% and specificity of 98% for proximal DVT, and 94% sensitivity and 75% specificity for distal venous thrombosis [1]. We performed this study to evaluate the findings of duplex scan in patients with clinical suspicion of DVT.

## Materials and methods

Two hundred thirty-nine consecutive symptomatic outpatients (59.2 ± 18.6 years, 164 female) with clinical suspicion of DVT (calf or thigh pain, limb swelling, tenderness, cyanosis, cellulites, venous stasis or joint pain) where evaluated by duplex scan, including iliac veins. Information on demographic characteristics and risk factors were collected using a structured questionnaire elaborated by Non-Invasive Methods Department of Brazilian Society of Angiology and Vascular Surgery. The data also included symptoms, site of DVT, normal or pathologic duplex scan, and other found pathologies. Measurements were taken with a high-resolution B-mode ultrasonography (Philips Medical Systems' Envisor C platform) with a broadband width linear array transducer L 3–12 MHz. Sonography and readings were carried out by trained and certified sonographers. Briefly, the vein compressibility, the presence or not of venous flow, the presence or not of venous thrombus and the response to distal compression maneuver were recorded.

### Statistical analysis

Results were expressed as percentages with 95% CI.

## Results

**Figure 1:**
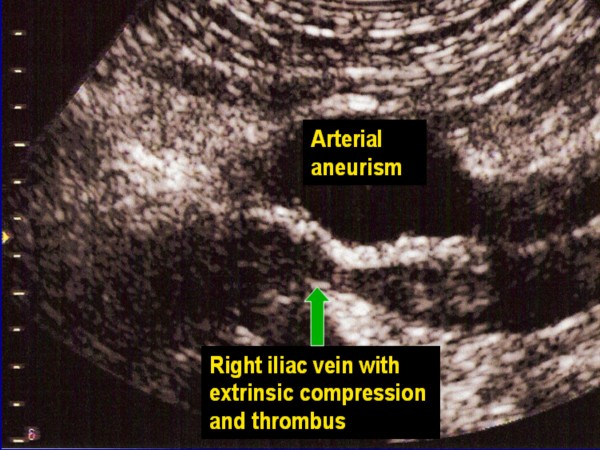
Extrinsic compression of iliac vein by arterial aneurism.

**Figure 2:**
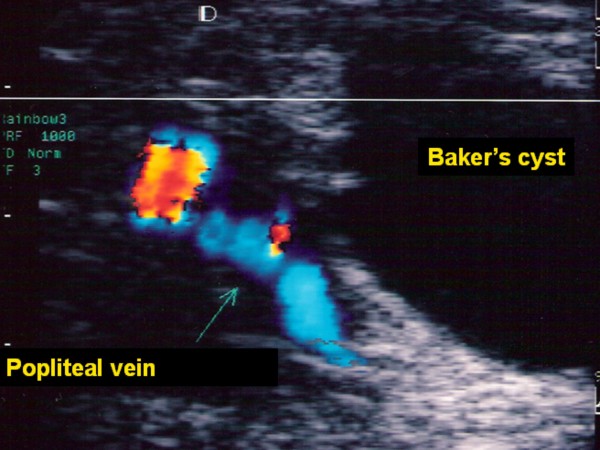
Baker's cyst.

**Figure 3:**
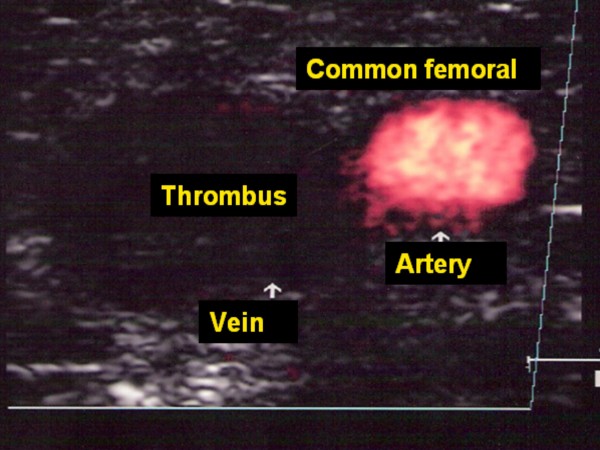
Thrombosis of common femoral vein.

**Figure 4:**
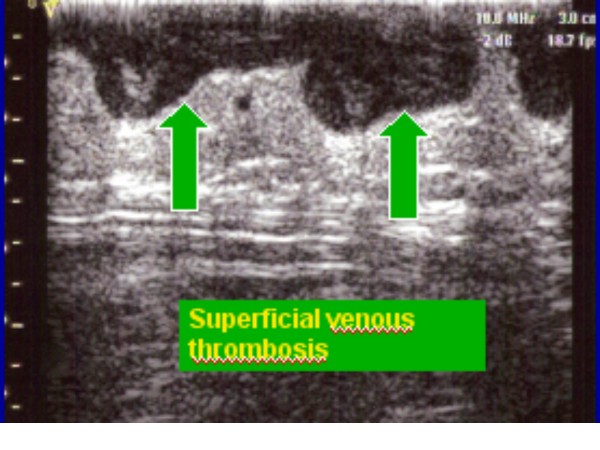
Superficial venous thrombosis.

**Figure 5:**
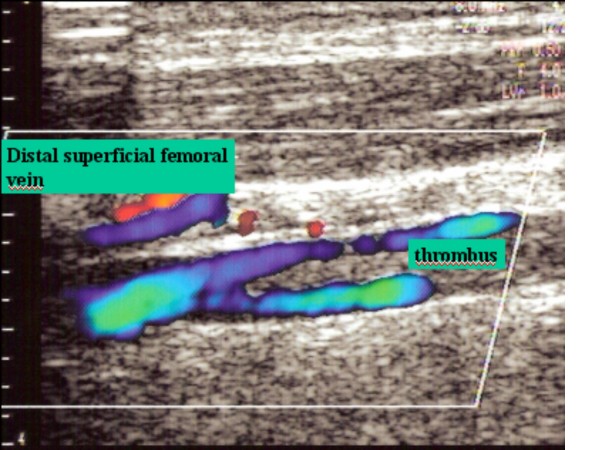
Distal femoral superficial vein with partial thrombus in vein lumen.

**Figure 6:**
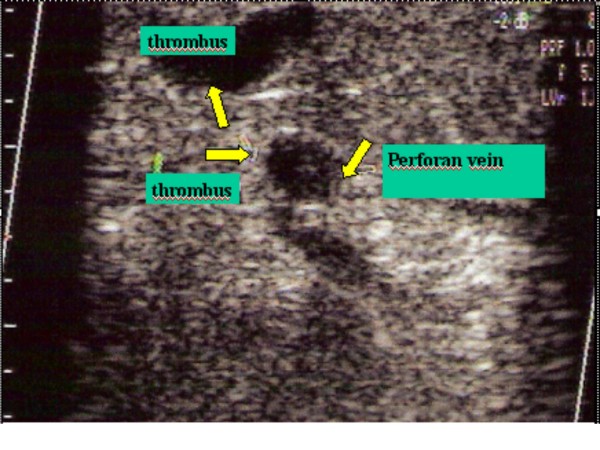
Thrombosis of perforan vein.

Clinical parameters indicating duplex scan are displayed on Table 1. According to symptoms, 101 (42.3%) were related to right lower limb; 113 (47.3%) to left lower limb; and 25 were related to both lower limbs. Forty-eight patients presented a normal duplex scan. Venous thrombosis was found in 117 patients (0.49; CI 0.43–0.55): 75 with DVT, 20 with both superficial venous thrombosis (SVT) and DVT, and 22 with SVT alone. Other pathologies were found in 74 patients. Among other pathologies, old thrombosis was most frequent (23 cases; 31%), followed by edema (18 cases; 24.4%) (Table 2 & Figures 1, 2, 3, 4, 5, and 6). Among patients with DVT the most involved veins were below the knee, followed up by popliteal vein, superficial femoral vein and common femoral vein. Among patients with SVT, in 20 (47.6%) there was progression to the deep venous system: in 9 (45%) by perforans veins; in 6 by saphenous-femoral junction (30%); and in 5 (25%) by saphenous-popliteal junction. We did not perform contrast venography after duplex scan. The decision about medical treatment and follow-up of each patient was made by the referring physician according to individual needs. But in our setting physicians generally request only the duplex scan without complementation for clinical suspicion of DVT and the management of patients is performed in accordance with the results of the duplex scan. Therefore, it was not possible to calculate the sensitivity, specificity and accuracy of this method in the present study.

**Table 1:** Clinical parameters for performing duplex scann

Clinical parameter	N – %
Limb swelling	201 – 84.1%
Calf or thigh pain	121 – 50,6%
Tenderness	15 – 6.2
Cyanosis	10 – 4.1%
Cellulitis	10 – 4.1%
Venous stasis	9 – 3.7%
Joint pain	5 – 2%

**Table 2:** Incidence of other pathologies

Other pathologies	N – %
Old thrombosis	23 – 31%
Edema	18 – 24.4%
Baker's cist	15 – 20.2%
Haematoma	6 – 8.1%
Inflammatory process	6 – 8.1%
Extrinsic compression	5 – 6.8%
Intramuscular liquid collection (haematoma or abscess or synovial fluid)	1 – 1.3%

## Discussion

In a systematic review [2], the incidence of DVT in the entire general population is approximately 5 per 10000 per year of which 2 per 10000 are idiopathic. An additional 1–2 per 10000 have a new DVT combined with pulmonary embolism. In pregnant women the incidence of venous thrombosis is 1 in 1000 – 20000 pregnancies [3]. Fast and accurate diagnosis of DVT allows for immediate treatment and improves clinical outcome. Chronic complications include recurrent thrombosis, post-thrombotic syndrome and chronic pulmonary hypertension [4-6]. In addition, in a recent cohort analysis of 4890 patients Huerta et al [7] found that an episode of venous thromboembolism is associated with a slight increased risk of myocardial infarction and increased risk of overall deaths during the first year after a venous thromboembolism episode. The modalities to diagnose DVT have improved substantially over the past decade. However, since DVT cannot be diagnosed solely by history and physical examination and requires specialist investigation, the role of duplex scan is crucial. Duplex scan can correctly diagnose deep and superficial venous thrombosis and can also precisely identify other pathologies such as Baker's cyst, haematoma, extrinsic compression and edema. In addition, with increasing frequency, physicians are requesting duplex scan to detect lower extremity DVT in the event of suspected pulmonary embolism, even in the absence of leg symptoms. In a study to determine the incidence of DVT in a high-risk group of ICU patients receiving DVT prophylaxis, a duplex scan was performed in 102 subjects [8]. Twelve patients receiving DVT prophylaxis were documented to have DVT by venous duplex scans. In patients without signs or symptoms of DVT, only two (3.6%) presented abnormal scans. The authors recommended that venous scans be performed only in patients with features suggestive of DVT or pulmonary embolism. In the present study, all patients presented symptoms. In addition, the symptoms due to other pathologies were very similar to DVT. These factors make the duplex scan very important in differential diagnoses. Duplex scanning has improved in precision and has gained popularity. It is safer than other invasive techniques, such as contrast venography, and also provides a more timely diagnosis and in a more efficient manner than most noninvasive techniques [9]. In a prospective, double-blind study, Killewich et al [10] found a sensitivity and specificity for duplex scanning relative to contrast venography of 85–95%. For deep venous thrombosis below the knee, Miller et al [11] found a sensitivity and specificity for duplex scanning relative to contrast venography of 85.2%–99.2%. In the present study we did not perform contrast venography after the duplex scan. The referring physician's decision to continue the investigation or not was based on the duplex scan results. However, as previously mentioned, in our environment the physicians only request the scan in the case of suspicion of DVT. Currently, it is not common to request a contrast venography after a duplex scan. Consequently, the definitions of sensitivity, specificity, accuracy, and negative and positive predictive values are not adequate in this study. As a non-invasive technique, it is the method of choice for high-risk patients. For example, in haemodialysis patients, with haemostatic disorders, duplex scanning could be performed safely [12]. In cancer patients, duplex scanning is the method of choice for the diagnosis of central venous catheter-related upper extremity deep venous thrombosis in symptomatic patients and for screening of asymptomatic thrombosis in this specific population [13]. However, some issues should be considered. In early and asymptomatic DVT, diagnosis by duplex scanning shows a decrease in accuracy. This is due to the fact that the fresh thrombus is not occlusive, has the same echogenicity as blood, and has a reduced consistency, therefore jeopardizing the compressibility test, the most sensitive test for DVT [1]. In these cases, duplex scanning should be performed 2–3 days later to confirm or exclude the diagnosis. In addition, the sensitivity and specificity of duplex scanning in vessels below the knee is not good. However, with improvements in echo machines and with meticulous technique, duplex scanning is highly accurate in diagnosing acute symptomatic deep vein thrombosis in lower extremities, thus invasive techniques are avoided in over 90% of the cases, even at the tibioperoneal level. There are no guidelines regarding the diagnosis of DVT, however some algorithms are adopted. In these algorithms, the duplex scan should initially be performed to accurately exclude or confirm DVT [4]. In the present study the duplex scan was the first method of choice for the referring physician to either confirm or exclude DVT and look for other pathologies. It is a convenient, safe and quick exam and most physicians prefer it to other methods when faced with patients presenting leg swelling.

## Conclusion

Deep venous and superficial venous thromboses were diagnosed in 49% of the cases. In 30.9% of the cases, the duplex scan presented other pathologies.

## Competing interests

The authors declare that they have no competing interests.

## Authors' contributions

AO participated in the study design. GJF participated in data collection. EAV participated in data collection. PSDS participated in data collection. LAVB wrote and oriented the manuscript. All authors read and approved the final manuscript.
